# HIV care engagement and ART adherence among Kenyan gay, bisexual, and other men who have sex with men: a multi-level model informed by qualitative research

**DOI:** 10.1080/09540121.2018.1515471

**Published:** 2019-01-22

**Authors:** Susan M. Graham, Murugi Micheni, Andrew Secor, Elise M. van der Elst, Bernadette Kombo, Don Operario, K. Rivet Amico, Eduard J. Sanders, Jane M. Simoni

**Affiliations:** a Departments of Medicine, University of Washington, Seattle, WA, USA; b Departments of Global Health, University of Washington, Seattle, WA, USA; c Departments of Epidemiology, University of Washington, Seattle, WA, USA; d KEMRI Wellcome Trust Research Programme, Kilifi, Kenya; e Department of Global Health, University of Washington, Seattle, WA, USA; f Department of Behavior and Social Sciences, Brown University School of Public Health, Providence, RI, USA; g Department of Health Behavior and Biological Sciences, University of Michigan School of Nursing, Ann Arbor, MI, USA; h Nuffield Department of Medicine, University of Oxford, Oxford, UK; i Departments of Psychology, University of Washington, Seattle, WA, USA; j Departments of Gender, Women and Sexuality Studies, University of Washington, Seattle, WA, USA

**Keywords:** HIV/AIDS, men who have sex with men, antiretroviral therapy, treatment adherence, social stigma, social discrimination, resilience

## Abstract

Gay, bisexual, and other men who have sex with men (GBMSM) are highly stigmatized and male–male sex is often criminalized in sub-Saharan Africa, impeding access to quality care for sexual health, HIV prevention, and treatment. To better understand HIV care engagement and antiretroviral therapy (ART) adherence among GBMSM in this context, a conceptual model incorporating sociocultural factors is needed. We conducted a qualitative study of barriers to and facilitators of HIV care engagement and ART adherence among Kenyan GBMSM, informed by a conceptual model based on an access, information, motivation, and behavioral skills (access-IMB) model, with trust in providers and stigma and discrimination as a priori factors of interest. We conducted 30 semi-structured interviews with HIV-positive Kenyan GBMSM, of whom 20 were taking ART and 10 had not yet initiated treatment. A deductive approach was used to confirm the relevance of basic concepts of the access-IMB model, while an inductive approach was used to identify content that emerged from men’s lived experiences. Access-related information, motivation, and behavioral skills appeared relevant to HIV care engagement and ART adherence, with stigma and discrimination appearing consistently across discourse exploring facilitators and barriers. Trusted providers and supportive family and friends helped many men, and resilience-related concepts such as selective disclosure of GBMSM status, connection to lesbian, gay, bisexual, and transgender (LGBT) organizations, self-acceptance, goal-setting, social identity and altruism emerged as important facilitators. Findings suggest a need to increase support from providers and peers for Kenyan GBMSM living with HIV infection. In addition, they point toward the potential value of interventions that provide opportunities to build or enhance one’s sense of community belonging in order to improve HIV care engagement and promote ART adherence for this vulnerable population.

## Introduction

Gay, bisexual, and other men who have sex with men (GBMSM) are a marginalized group in sub-Saharan Africa, with a disproportionately high HIV prevalence relative to men in the general population (Smith, Tapsoba, Peshu, Sanders, & Jaffe, 2009; Beyrer et al., 2012). African GBMSM face systemic stigma, discrimination, and criminalization, which constitute important barriers to accessing care and prevention services (Fay et al., 2011). Among HIV-positive GBMSM, stigma related to same-sex behavior augments HIV-related stigma, further impeding care engagement (Lane, Mogale, Struthers, McIntyre, & Kegeles, 2008; Sharma et al., 2008). For example, Cloete et al. reported that HIV-positive GBMSM in South Africa experienced higher levels of discrimination than did HIV-positive heterosexual men (Cloete, Simbayi, Kalichman, Strebel, & Henda, 2008). Greater exposure to health services may result in more adverse experiences; for example, in a survey of GBMSM living in Malawi, Namibia, and Botswana, being treated for HIV was associated with a greater odds of fearing to seek health care services, ever having been denied services on the basis of sexuality, and having been blackmailed due to sexuality (Fay et al., 2011). Little is known about how GBMSM are affected by the pervasive stigma and discrimination in many African healthcare settings and the ways in which men may overcome barriers to engage successfully in care. A better understanding of these barriers and facilitators is urgently needed to inform the development of effective HIV prevention and care interventions tailored for African GBMSM.

Our work was guided by the Information, Motivation, and Behavioral skills (IMB) model (Fisher, Fisher, Amico, & Harman, 2006), which has been validated in African settings and used to identify barriers and facilitators of ART adherence in the general population of African adults (Simon, Altice, Moll, Shange, & Friedland, 2010; Croome, Ahluwalia, Hughes, & Abas, 2017). Due to the importance of health care access for African GBMSM, we selected an access-IMB model adapted by Starks et al. as our conceptual framework (Starks et al., 2008). This model suggests that four components are necessary for HIV care engagement and consistent ART adherence:

1. access to trained providers and medications;
2. information on ART dosing, potential side effects, and the potential for developing resistance if non-adherent;
3. motivation to maintain health by taking a daily medication; and
4. behavioral skills such as cues to pill-taking, refills and appointment attendance.

This model was depicted inside a box to indicate the enabling environment that a trusted provider could create, within a larger box that represents the pervasive stigma and discrimination that impacts all model components. In the present qualitative study, we aimed to identify barriers and facilitators of HIV care engagement and ART adherence among Kenyan GBMSM and inform the development of a more complex and tailored conceptual model, as part of a larger study of an ART adherence support intervention for Kenyan GBMSM living with HIV infection.

## Methods

### Participant recruitment

Between June 2013 and February 2014, HIV-positive GBMSM were identified through peer outreach and referrals from local GBMSM-friendly providers. Men were eligible to participate if they were assigned male sex at birth, 18 years or older, lived or worked in the Malindi, Kilifi, or Mtwapa communities, had engaged in manual, oral, anal sex with a man during the past 12 months, had documented HIV-1 infection, and were able to communicate in Kiswahili or English. We included both ART-experienced and ART-naïve men to ensure that a range of HIV care experiences was represented. In addition, purposive sampling was used to ensure diversity with respect to age, educational background, employment history, and engagement in care. Recruitment and interviews continued until theoretical saturation was attained (i.e., No new concepts were emerging from new interview data), as determined by immediate review of post-interview notes and ongoing discussion between the interviewer and co-investigators.

### Procedures

Based on our conceptual model, we developed a topic guide that explored participants’ personal background, HIV testing history and diagnosis, medication history, disclosure issues, HIV knowledge, motivation, medication-related skills and self-efficacy, ART adherence (or co-trimoxazole adherence if not yet taking ART), problems with access or other barriers to successful engagement in care, facilitators of care engagement and ART adherence, past interactions with providers, and proposed elements of an ART adherence intervention specifically tailored to GBMSM.

All study procedures were conducted in private space at one of four HIV care clinics on the Kenyan coast between Malindi in the north and Mombasa in the south. After written informed consent was obtained, participants completed a brief sociodemographic questionnaire. Interviews were conducted by a trained interviewer fluent in English and Kiswahili (MM) following the topic guide. All participants but one consented to digital recording; detailed notes were taken for this participant instead. Interviews lasted approximately 90 min on average. Participants were compensated 350 Kenyan shillings (≈$3.75) for their time and transport costs, in accordance with local standards at the time. The interviewer wrote a brief summary of each interview immediately afterwards and shared this with the team. Two research staff transcribed recordings verbatim and checked all interview audio files for accuracy before translating any Kiswahili content into English.

### Analytic methods

StataSE version 14 (StataCorp LLC, College Station, TX) was used to compile descriptive statistics on study participants. Data transcripts were iteratively coded to identify themes within each content area. Initial codes were developed from the interview guide, field notes, and multiple readings of the transcripts. Using a deductive approach, two members of the research team (MM and AS) read and independently coded text segments relating to key concepts from the access-IMB model in QSR NVivo software version 10 (QSR International, Inc., Burlington, MA, USA). Using an inductive approach, the two coders also identified themes that were not accounted for in the access-IMB model and thus not originally in the coding dictionary. These themes were added to the coding dictionary, with the concurrence of the Principal Investigator (SMG) and team members expert in qualitative research (DO and EMV). One senior co-investigator (DO) reviewed a set of coded transcripts in order to assess the consistency of the approach. After all coding was completed, SMG, MM, and AS abstracted quotes coded for key constructs in the conceptual model and for additional themes or concepts that emerged during analysis.

The entire team reviewed the abstracted quotes for relevance to HIV care engagement and ART adherence, the salience of the content for Kenyan GBMSM, and “fit” with our conceptual model constructs. Using the heuristic schema of Ware, Wyatt, and Bangsberg (2006), we asked four analytic questions about the model validity and relevance: (1) Are the model’s basic concepts relevant in the setting? (2) Are all basic concepts important to the setting represented? (3) Are the meanings of the model’s basic concepts accurate in the setting? and (4) Does the model capture the complexity of adherence in this setting? Based on answers to these questions, we developed a more complex, situated final model that incorporates additional concepts and contextual factors.

### Ethical approvals

The study protocol, informed consent documents, and interview topic guides were reviewed and approved by the Ethical Review Board of the Kenya Medical Research Institute and the Human Subjects Division at the University of Washington.

## Results

### Study population

Thirty in-depth interviews were carried out before saturation was reached: 10 from ART-naïve men and 20 from ART-experienced men, of whom 2 had discontinued therapy. Participants came from 10 different ethnic groups and lived in 15 different villages or neighborhoods along the Kenyan coast. Characteristics of these men are included in Table 1. Median age was 31 years (range, 19–51 years). The median years of education was 8 years (range, 4–14 years). Most participants (60%) identified with a gay or homosexual orientation, while some identified as straight (7%) or bisexual (7%) and others (26%) chose a Kiswahili or other term for their identity. One participant identified as female but included herself in the “MSM” umbrella. The median duration of ART was 5 years (range, 1–10 years) among ART-experienced men.

**Table 1.** Sociodemographic characteristics of participating men.

Characteristic	*N* (%) or median (range)
Age (years)	31 (19–51)
Time at current residence (years)	22 (1–41)
Education (years)	8 (4–14)
Employment type	
Formal	3 (10)
Casual	5 (17)
Self-employed	16 (53)
Unemployed	6 (20)
Religion	
Catholic	5 (17)
Protestant	8 (27)
Other Christian	3 (10)
Muslim	13 (43)
None	1 (3)
Marital status	
Single	12 (40)
Married, monogamous	2 (7)
Married, polygamous	1 (3)
Separated or divorced	9 (30)
In steady relationship	6 (20)
Sexual orientation	
Straight	2 (7)
Gay	15 (50)
Homosexual	3 (10)
Bisexual	2 (7)
None of these	4 (13)
Other	4 (13)
Ever had a female sex partner	20 (67)
Ever paid for sex	25 (83)
Has own toilet or latrine	11 (37)
Has electricity at home	22 (73)
Has piped water at home	24 (80)
Has a radio at home	25 (83)
Has a television at home	16 (53)
Has a cell phone	27 (90)
ART status	
Never started	10 (33.3)
Started but not taking now	2 (6.7)
Taking now	18 (60.0)
Duration on ART (years)^a^	5 (1–10)

^a^Among 20 men who had initiated ART.

### Themes

Many themes emerged from these interviews that reinforced the relevance of the basic access-IMB model components: access to care and basic needs; information about HIV and ART, personal motivation (e.g., Beliefs about ART efficacy, wanting to be healthy), and the skills needed to engage in care and adhere to ART. Examples of these themes, which have been commonly reported in African settings for many different types of patients, are summarized in Supplemental Table 1. In addition, an inductive coding approach identified constructs that were not well developed in our model but were particularly salient for Kenya GBMSM. These themes centered on the topics of *provider competence, stigma and discrimination, social support, and resilience*. Resilience, described by Herrick et al. as coping with negative experiences and avoiding negative trajectories (Herrick, Stall, Goldhammer, Egan, & Mayer, 2014), was a particularly important factor facilitating men’s motivation, not only for care engagement and ART adherence, but for living a healthy and fulfilling life. In the next section, we present participants’ quotes to illustrate how they described these emergent themes. Quotes are followed by information about participant age, ART status, and sexual orientation.

*Provider competence* in sexual health was lacking. Men complained about a lack of tailored information and services, with many providers assuming that men had sex with women only:

I went to the [Comprehensive Care Clinic] but I was not very comfortable … there you meet straight people, yet you want to talk about yourself. You are being asked about your wife, girlfriend … there was so much of these questions. I was afraid “how am I going to tell this person that am gay?” yeah so at least he will understand me but I decided to keep it privately and said “I had unprotected sex with a woman whom I did not know (her status) and I later found out I am HIV positive”. (35-y.o., ART-experienced, gay)

As a result, men were selective about which providers they saw:

If I went to a health facility the moment I meet you I can tell how homophobic you are or how friendly you are … I cannot access health care where there is stigma or a place where they are not sensitive to sexuality issues. (22-y.o., ART-naïve, gay)

No, I don’t tell my secret [when I go to a health facility]. Whoever is in a position to know my secret he/she will but whomever I don’t want to know he will not. (37-y.o., ART-naive, “Basha” (insertive sexual partner))

As posited by our initial conceptual model, a relationship with a *trusted, non-stigmatizing provider* was important for many men:

… You could not go around looking for an MSM doctor. It’s just the straight person there and if he is sincere and follows his work ethics and is not discriminatory, then he will not have a problem working with the MSM … Personally, if I like you and I can open up to you, I will tell you everything and it is not because there is anything going on, but because I trust you. I feel like this is the doctor that I am comfortable with – this other one I am not comfortable with. (35-y.o., ART-experienced, gay)

In terms of *stigma and discrimination*, men were afraid of exposing themselves to gossip about their sexual behavior or lifestyle, whether from providers or others in the community:

I didn’t want to be a subject to be talked about because already, just the life style. Some people suspected that I was gay so to add HIV on it and at that time HIV being a gay disease … you hear them say “that is the price of being gay”. (37-y.o., ART-experienced, gay)

This *stigma and discrimination* contributed to mental health or substance abuse problems reported by participants:

Alcohol is our [GBMSM] thing … it makes us forget everything, forget about “so and so will say, so and so is looking at me, so and so will discuss me” yeah! … Don’t just see someone drinking (alcohol) and assume he is happy or he has money, NO! There is something disturbing him psychologically. (35-y.o., ART-experienced, gay)

Unfortunately, stigma often led to nondisclosure of HIV status and an inability to mobilize *social support* from family or friends:

I am a man, but am doing what is against family values and even religious values. As a man, I am expected to be heterosexual. However, my feelings are to the contrary. Therefore if I tell my mother or my sisters that I am positive … don’t you think I will be calling for abuses? (28-y.o., ART-experienced, gay)

However, in many cases, family members or friends did provide valuable *social support* when men disclosed:

I disclosed to my sister about my HIV status last year. I was encouraging her to swallow her drugs: “I don’t want to hide anymore. We are all going through the same thing”. She said to me: “It’s good that you have shared about it, please never ever risk your life”. This gave me courage, that people still care about me. (40-y.o., ART-experienced, gay)

I don’t need money because I know I am going to leave everything in this world. All I need is company, and people to talk to. (33-y.o., ART-experienced, gay)

In addition to support from providers and family, many men spoke about the importance of their *social connection* to the lesbian, gay, bisexual, and transgender (LGBT) community:

We had a support group that brought the MSM together … we used to help one another even if we did not have that much. We were there for each other … An MSM with another MSM will socialize [better] than with a straight person … The important thing considering the MSM the way they live, they need somebody to listen to them. (35-y.o., ART-experienced, gay)

It’s you straight people who imagine that MSM are afraid to come out. We have an [out] health officer for PEMA Kenya [LGBT organization], we have an [out] health officer for GALCK [another LGBT organization] … They are very ready. (25-y.o., ART-naïve, gay)

However, *resilience* appeared critical as an adjunct to provider trust and social support. As helpful as LGBT community support was, men’s resilience appears rooted in their own self-acceptance and acknowledgment of their self-worth:

My drive is from what I have gone through and self-acceptance. I know that my health is my responsibility and that with counselling and proper use of my meds I have hope and I see that life can continue as normal. (31-y.o., ART-experienced, “Basha” (insertive sex partner))

Another man’s resilience was based in his recommitment to securing a healthy, happy, and fulfilling life:

What made me continue with my medication is because I wanted to live a healthy life. I wanted to be somebody. I wanted to be back on track to do what I used to do in a happy lifestyle and not falling sick most of the time. (35-y.o., ART-experienced, gay)

Yet another man’s resilience was demonstrated by the ambition to start his own business:

I hope to get employed, rather than idling. I won’t have time to think and worry of what people would say, or feel withdrawn, when I’m engaged. If I have a business, then it will keep me busy – my business shall be my only other partner. (33-y.o., ART-experienced, gay)

Men’s resilience did not operate solely through a renewed and more positive focus on the self. Consistent with the communal and collectivistic culture of Kenya, many men found helping others to be an important source of resilience:

I was also trained as a community health worker and I was reaching to the general community there … It was good … we were six of us and we were educating them on proper hygiene, treating water, recording of new births, making referrals for TB and HIV to [the] dispensary. (36-y.o., ART-naïve, straight)

Even as we talk there is someone I have brought [to this clinic] … Last week I brought him … and he was tested for HIV where he tested positive. So now I am helping him; he has already had TB screening and now I left him at the clinic opening a file … I like helping others. I don’t like seeing others being caught off guard. (40-y.o., ART-experienced, “MSM”)

### Final conceptual model

**Figure 1.**
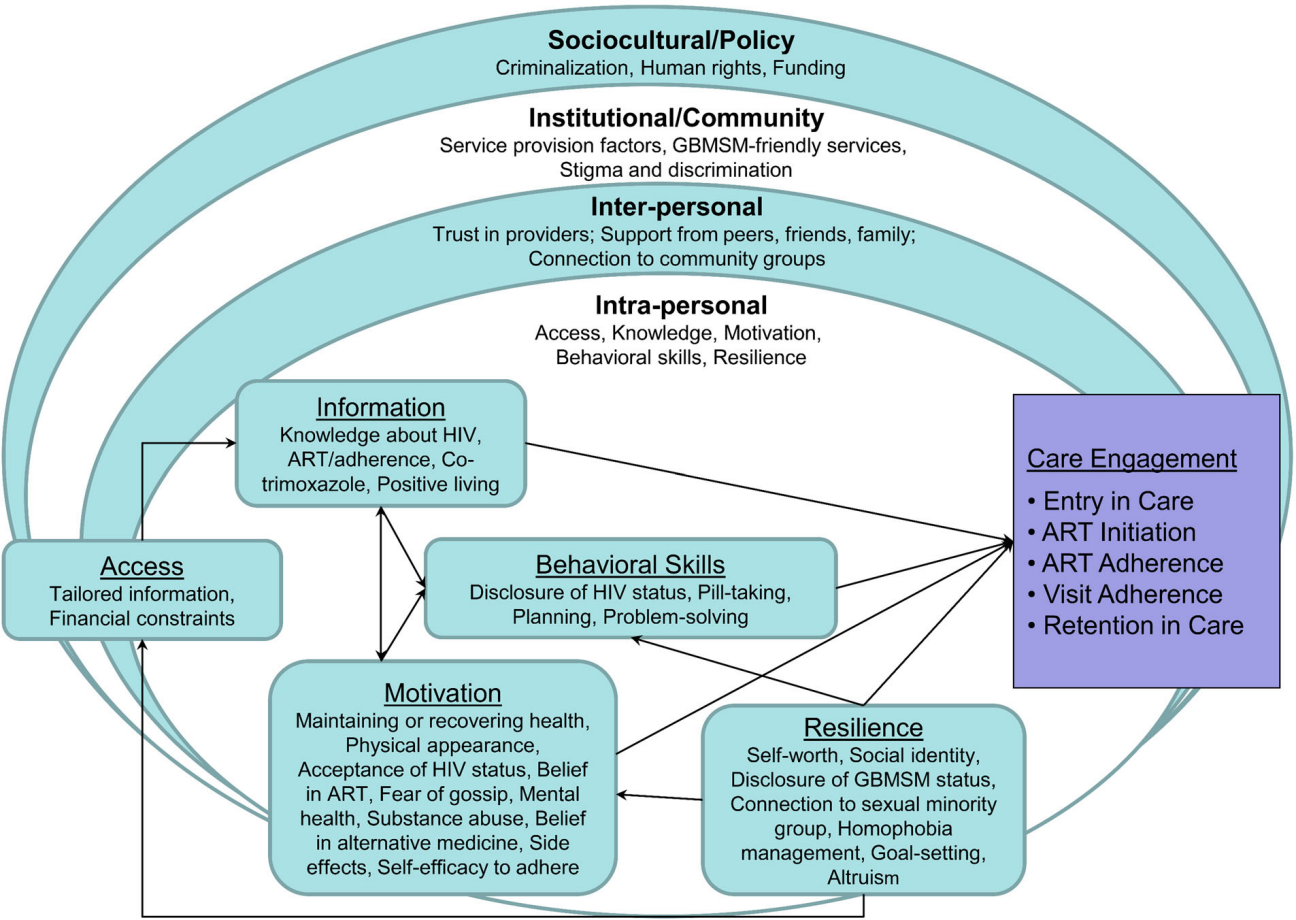
Final situated access-IMB model of HIV care engagement and ART adherence among Kenyan GBMSM.

Based on our qualitative inquiry, we determined that our model of HIV care engagement and ART adherence for Kenyan GBMSM needed to be more complex and multi-level. This expanded model (presented in Figure 1) incorporates themes related to the access-IMB model that were identified using a deductive approach, as well as themes that inductively emerged from the data. These themes are depicted according to a socioecologic model with intra-personal, inter-personal, and institutional/community levels. Although men did not specifically talk about laws, rights, and funding to support GBMSM programming, we added a sociocultural/policy level that included these factors, which provide critical context underlying many of the barriers identified. In addition, our final model adds resilience as an important influence that emerged from our inductive analysis. Resilience-related factors highlight the inner resources and strength that many Kenya GBMSM living with HIV infection are able to draw upon as they strive to maintain health in a rights-constrained setting.

## Discussion

Findings from ART-experienced and ART-naïve GBMSM in the present study highlight the marginalized and vulnerable context of this population, for whom tailored information and services are largely unavailable in sub-Saharan Africa (Smith et al., 2009). We found that trust in providers was a key factor helping GBMSM to engage in care and adhere to treatment in the setting of systemic stigma and discrimination. However, some men fell out of care when their preferred provider became unavailable, and others experienced tensions with providers whom they found rigid or stigmatizing. Most men felt uncomfortable discussing sexual risk behavior with providers and preferred that interactions focus on adherence and other aspects of health care instead. As we considered implications for intervention development, we concluded that providers need training in male sexual health and in patient-centered care. In the development of our intervention, we called *Shikamana* from the Swahili term for “to form a bond or stick together” (Graham et al., 2017), we made adherence the primary focus for provider counseling, since virologic suppression not only prevents morbidity and mortality but also greatly reduces sexual transmission risk (Cohen et al., 2016).

Resilience emerged as an important individual-level construct influencing HIV care engagement and ART adherence in this population. While we considered that peer support would likely be important, we did not explicitly include this or other resilience-related concepts in our original conceptual model because we were unsure of the role these concepts would play. We found that several study participants had had at least some training through local HIV prevention and care initiatives and were interested in helping others in their communities. For many men, becoming connected to others who were living with HIV or who identified as gay or bisexual, particularly in the context of emerging local LGBT organizations, played an important role in their ability to cope with HIV infection. Such connections have been found important in other minority communities in which stigma plays an important role. For example, peer connectedness had a positive association with condom use among multiracial young MSM in New York City (Mutchler et al., 2011). In addition, peer support was associated with recent HIV testing among young Black MSM in the US South (Scott et al., 2014). Relatively little research has been done on the role of resilience in promoting care engagement and ART adherence among GBMSM living with HIV in settings with systemic stigma and discrimination. Our findings indicate that resilience among GBMSM individuals and communities may be especially important to tap into, in order to improve HIV prevention and care services and outcomes for GBMSM (Graham & Harper, 2017).

While the initial conceptual model that guided this work included a priori factors we considered important, we had not explicitly started with a situated model. A situated-IMB model was originally proposed as an explanation of “care initiation and maintenance” for chronic medical conditions (sIMB-CIM), where each of the core IMB model constructs of information, motivation, and skills were “situated” into the socioecological context of negotiating care (Rivet Amico, 2011). Applied to HIV care engagement, the sIMB-CIM model emphasized affective components, competing priorities, and the role of stigma and privacy as relevant across the core constructs. This model has been used in qualitative work with inner-city patients accessing HIV care in the Bronx, NY (Smith, Fisher, Cunningham, & Amico, 2012). Using inductive analysis of emergent themes, Smith *et al* identified a number of factors influencing retention in care, including acceptance of diagnosis, stigma, HIV cognitive/physical impairments, and global constructs of self-care, and confirmed that emergent themes aligned strongly with the situated-IMB model’s constructs (Smith et al., 2012). A situated-IMB model has particular appeal as an approach both to characterize health behaviors and to develop relevant interventions. In the current study, we determined that the core constructs of our access-IMB model are important among GBMSM in coastal Kenya. We then expanded upon the basic access-IMB model to situate it in a socioecologic setting including contextual factors that influence the access-IMB constructs. These same contextual factors influence resilience, which we added as a construct to capture the personal resources men draw on to cope with stigma and discrimination. We propose that future research formally test this model to predict behavior in longitudinal cohorts and to develop and test structural-, community/institutional-, or individual-level interventions to reduce health disparities faced by GBMSM in Kenya and other rights-constrained settings.

Globally, the HIV epidemic remains uncontrolled among GBMSM, in large part due to stigma, discrimination, and inadequate access to tailored services for prevention and care (Ayala & Santos, 2016). We found in our interviews with Kenyan GBMSM living with HIV infection that service provision factors and clinic-based support groups were either weak facilitators or barriers to men’s care engagement, indicating that more tailored and patient-centered services are required to address men’s needs. Decriminalization of male–male sex and advocacy for equal rights and protections for GBMSM are urgently needed in order to optimize the response to the HIV epidemic globally, in line with WHO recommendations (Organization, 2011). While Kenyan GBMSM are benefiting from inclusion by the National AIDS and STI Control Programme as a key population for HIV prevention and care programming (Sanders, Jaffe, Musyoki, Muraguri, & Graham, 2015), punitive laws in Kenya and many other African countries have been associated with implausibly low or absent GBMSM population size estimates that hinder access to care and impede progress in containing the HIV epidemic (Davis, Goedel, Emerson, & Guven, 2017).

There are several limitations to this study. First, our interviews relied on retrospective recall of experiences, and some of the details may be inaccurate. Second, our topic guide specifically addressed access-IMB model constructs and so participants were not blind to our hypotheses that these would be important. Third, as it is very hard to reach GBMSM in Kenya who are not out to others, our participants likely represent a biased sample with stronger connections to peer training and LGBT organizations. Our results are not generalizable to hidden GBMSM or to men who do not speak English or Kiswahili, the two national languages of Kenya. Fourth, as KEMRI has trained many health providers in the study area in GBMSM sexual health, our study is not representative of areas with a total lack of trained providers. Finally, although one of our participants identified both as an “MSM” and a transgender woman, our study focus was on GBMSM and our results are not representative of the experiences of transgender women in Kenya. The strengths of the study include the detailed interviews conducted that reflect men’s experiences as GBMSM living with HIV in Kenya, the range of ages and sexual orientations of men who participated, and the sampling of participants from several different locations along the Kenyan coast so that men in both urban and rural settings were included.

While acknowledging the above limitations, our data support the utility of a situated access-IMB (access-sIMB) model as a potential framework for understanding theoretical and contextual determinants of HIV care engagement and ART adherence for Kenyan GBMSM living with HIV infection. Our final situated model comprises factors at the individual, inter-personal, institutional/community and sociocultural–policy levels that should be addressed in intervention development and were included in our work to develop an ART adherence support intervention for this population (Graham et al., 2015). Despite immense challenges due to stigma and discrimination, men’s engagement in care and adherence to ART are facilitated by access to tailored information and GBMSM-friendly services as well as men’s own resilience and support from friends, family, peers, and LGBT organizations. Although men do often find providers they trust, providers in rights-constrained settings need training and structural support to provide non-stigmatizing, patient-centered care. Interventions to promote care engagement and increase adherence for this population should address the multiple determinants identified in our model.

## Supplementary Material

Supplemental Material
