# Supplementary material for: HIV care engagement and ART adherence among Kenyan gay, bisexual, and other men who have sex with men: a multi-level model informed by qualitative research
Source: AIDS Care. 2019 Jan 22;30(Suppl 5):S97–S105. doi: 10.1080/09540121.2018.1515471 (PMC6430645; doi:10.1080/09540121.2018.1515471)
Supplement: Supplemental Material [file CAIC_A_1515471_SM6981.docx]

**Supplemental Table 1. Representative Quotes on Relevance of Basic Components of the Access-IMB Model among Kenyan GBMSM, by Emergent Theme**

| ***Access***  ***Barriers***  ***Poverty and food insecurity***   - *I get stressed but it’s not related to my health, it’s just other forms of stress…like where will I get money tomorrow, the landlord wants his money.* – 25-y.o., ART-naïve, gay - *[When I was on the streets], I tried [taking my ART without food] and it was really difficult. I was not using them: why lie? Because the experience was really bad.* – 51-y.o., ART-experienced, bisexual   ***Drug stock-outs***   - *I hear that an individual who is used to taking the HIV medications is likely to die if he runs out of the drugs…Sometimes the government runs out of drug supply, and it affects so many people.* – 29-y.o., ART-naïve, homosexual |
| --- |
| ***Information***  ***Facilitators***  ***Knowledge about ART, adherence, positive living, co-trimoxazole***   - *Being diagnosed HIV positive does not mean the end of life because meds are there as long as you take them as required*. – 40-y.o., ART-experienced, “Basha” (insertive sex partner) - *I later on realized that, majority of those whose condition has gone down, are people who are still in denial, or people who don’t adhere well to their medications: today they take, tomorrow they skip, or they stop for three months.* – 33-y.o., ART-experienced, gay - *I am also using Septrin and according to what I was informed about it,… when you are on Septrin it limits the chances of getting pneumonia or if you got it, it is much easier to treat it.* – 31-y.o., ART-experienced, homosexual   ***Barriers***  ***Lack of knowledge about ART, adherence, positive living, co-trimoxazole***   - *What I had heard is that HIV is a very bad disease; when you get infected with HIV that is the end of your life.* – 31-y.o., ART-experienced, homosexual - *I had no information; I knew there is AIDS and that was it … I didn’t even know about condoms…I knew AIDS kills and I never thought that there is high risk of infection in anal sex.* – 34-y.o., ART-experienced, homosexual |
| ***Motivation***  ***Facilitators***  ***Maintaining or recovering health and physical appearance***   - *I see these meds as my well-being. They are the ones that made me recover, so when I remember where I have come from and where I am today, I see these meds as my well-being.* –31-y.o., ART-experienced, straight - *He [the counselor] really encouraged me. He even took me to [the General Hospital] and he told me ‘you see these people in the ward, if you don’t take care of yourself you will get to this stage’. You see a grown-up person in diapers! I was so afraid.* – 24-y.o., ART-experienced, gay - *I didn’t [miss a single pill]. I wanted that skin to clear so I didn’t stop. I took [ART] for all those seven months but when I saw in the seven months I had improved and I had bought a cream and my skin was soft.* – 24-y.o., ART-experienced, gay   ***Acceptance of HIV status***   - *I can tell you malaria kills more than HIV and something like cancer is not curable. But we are proud because HIV we have some drugs that we can take and continue living. So I don’t see why should someone continue to hide or refuse to accept his status because he is HIV positive. Stigma is not good.* – 51-y.o., ART-experienced, bisexual - *I never imagined that I would one day become infected, given the fact that I used to teach people how to protect themselves and on how to stay with infected individuals in the community. But now it’s me, and I don't see it as a big deal because I am a peer educator. I would often go for trainings, and the knowledge that I acquired from these trainings has helped me to accept my status, and live positively, to my last day as God wishes.* – 33-y.o., ART-experienced, gay   ***Belief in ART***   - *I believe in [ART] for sure. As a matter of fact, whenever I am consistent [with taking ART], I improve a great deal and add a lot of weight to an extent that people wonder what has happened. –* 35-y.o. ART-experienced, “bottom” - *I am where I am today where I never thought I would be and it’s because of HIV medications. If my friends would have managed as I did, then they would be here today. If they would have had access we would be having them around to date.* *–* 35-y.o. ART-experienced, gay   ***Self-efficacy to adhere***   - *When I remember where I have come from and where I am today, I see these meds as my well-being. We were taught not to miss even a single dose. Even when I travel I carry my meds for three months.* – 31-y.o., ART-experienced, straight - *I cannot accept to die because I failed to use my meds ... to me it will never happen that way.* – 51-y.o., ART-experienced, bisexual - *I don’t even need anyone to remind me and I am grateful. For me it’s just automatic in my mind. No matter how engaged I am I always have to remember my pills when it is time. As a matter of fact, I am always very anxious as from 8 o’clock until I take my pills then I feel normal again.* – 35-year-old, ART-experienced, “bottom”   ***Barriers***  ***Fear of gossip***   - *You know many people are afraid of going for the test, I can even come with one of them and I tell you ‘this one is afraid to test because he feels that the person testing him will gossip about him or when he finds that he is positive he will undermine him or discriminate against him’* – 22-y.o., ART-naïve, trans woman - *I do get many [peers] who express interest in accessing care but they are concerned about being gossiped, and come to me for guidance; ‘Are you sure that the doctors won't talk about my life, or tell everyone about my condition?’.* – 33-y.o., ART-experienced, gay   ***Mental health challenges***   - *I had thought ‘I will get better on beginning the HIV medications, but know I am just weak. I feel… tired, I don’t feel anything, am just angry.’ I was about to give up and say ‘ah me I won’t take them’* – 35-y.o., ART-experienced, gay - *Sometimes I sit and really think about it … especially when I don’t have anything. I just have so many thoughts…I begin to feel like ‘what did I do wrong that I have to go through this.’* – 41-y.o., ART-experienced, “Shoga” (effeminate man)   ***Substance abuse***   - *Those [patients] who are forgetful are mainly the people on drugs. I have friends who take Miraa on a daily basis… when you take Miraa you are likely to forget.* – 31-y.o., ART-experienced, straight - *I do remember [to take my meds] as long as I am not drunk. –* 36-y.o., ART-experienced, bisexual   ***Belief in alternative or traditional medicine***   - *Such people are still in denial… so you see such a person will be the one swayed off by being told about Dynapharm meds [herbal medicines] and he stops taking the ARVs ... because he has not accepted himself.* – 40-y.o., ART-experienced, “MSM” - *Some of these companies are creating a mess because you find there are patients on ARVs and when they get herbal medicine they stop using the ARVs. You may find some patients are using [immune] boosters. There are companies that are selling these boosters.* – 22-y.o., ART-naïve, gay   ***Side effects***   - *[I was] feeling dizzy, nausea, inflamed and painful lips compounded with lack of appetite. [But] I [still] used them [HIV medications].* – 31-y.o., ART-experienced, “Basha” (insertive sex partner) - *At the beginning I felt ... and I had been informed that it will happen i.e. feeling overly sleepy, when I had just started ARVs. I didn’t stop ... I had just started so how could I have stopped, yet I had been told my life depended on them.* – 40-y.o., ART-experienced, “Basha” (insertive sex partner) |
| ***Skills***  ***Facilitators***  ***Pill-taking cues***   - *There is a TV program that I watch at 9.00 PM. TV stations in Kenya also air news at 9.00 PM. I pick my container and take my pills, when 9.00 pm news are about to start.* – 40-y.o., ART-experienced, gay - *I have set my phone alarm, and also, sometimes my close friends call to remind me; "Your time is ready, please take your medicine". The friend is infected, and also on drugs. We take our drugs at the same time; we are like treatment buddies.* – 33-y.o., ART-experienced, gay   ***Planning ability***   - *If I know I am going to town, before I leave I make sure that I load my dose for the day in this bag [pointing to his bag]. So if I stay late and my pill time finds me in town, I will just go to my bag and get my pills.* – 35-y.o., ART-experienced, “bottom” - *I make sure I often look at my clinic card and mark the date. I know that every month … because I used to go for refills on the 24th of every month but now it changed to 28th so I am always very keen to mark that date and I still confirm with my clinic card.* – 31-y.o., ART-experienced, straight   ***Problem solving***   - *It’s good to be open, when you are asked something you shouldn’t lie because it’s for your benefit… So it’s you to tell him that ‘the pill time you have given me isn’t appropriate for me because I work at this time, please give me another time.’ –* 24-y.o., ART-experienced, gay - *Last month I had gone to Busia and my meds got finished when I was there so I went to the nearest VCT and explained to them, ‘here are my tickets, I travelled from Malindi and there are meds that I use and I have run out of them. I had expected that I will go back in time but what I had come to do I have not finished, so I am requesting if you can give me meds for the days remaining.’* – 51-y.o., ART-experienced, bisexual   ***Barriers***  ***Difficulty taking pills***   - *What was difficult was when its pill time and maybe I am with friends who aren’t aware of my status and I want to take those drugs. You may find someone who is keen on knowing where you are going ... so sometimes I was forced to take it from the toilet and wash it down with water I am holding with my hands and I would walk out as if I had gone to relieve myself.* 51-y.o., ART-experienced, bisexual - *They are many pills, they are a burden, where to keep them ... it was a burden. Even now that I am used to them, taking them is still difficult.* – 30-y.o., ART-experienced, gay   ***Failure to plan***   - *In the past week …. I have missed three morning doses..Sometimes I leave home in the evening and fail to return or return very late.* – 36-y.o., ART-experienced, bisexual   ***Non-disclosure of HIV status***   - *I don’t mind about being an MSM, but for being HIV+ ... you know many people do not know; people are discriminated for being HIV+. You know people lack information, there are those that imagine when you touch them you will infect them.* – 22-y.o., ART-naïve, trans woman |
